# Paracervical block during vaginal natural orifice transluminal endoscopic surgery reduces postoperative pain and analgesic consumption: a retrospective cohort study

**DOI:** 10.20452/wiitm.2025.17979

**Published:** 2025-09-26

**Authors:** Merve Genco, Mehmet Genco, Feyza Azmak Çinaz, Semih Çinaz

**Affiliations:** Department of Obstetrics and Gynecology, Kayseri State Hospital, Kayseri, Turkey; Department of Obstetrics and Gynecology, Kayseri City Hospital, Kayseri, Turkey; Department of Department of Anesthesiology and Reanimation, Akhisar State Hospital, Manisa, Turkey

**Keywords:** laparoscopy, paracervical block, postoperative pain, tubal ligation, vaginal natural orifice transluminal endoscopic surgery

## Abstract

**INTRODUCTION:**

Vaginal natural orifice transluminal endoscopic surgery (vNOTES) for bilateral tubal ligation may cause notable early postoperative pain, leading to opioid use. Paracervical block (PBC) could support analgesia within enhanced recovery after surgery (ERAS) pathways.

**AIM:**

The aim of this study was to evaluate whether the use of PBC improved postoperative pain scores, reduced analgesic requirements, and affected short-term sexual function.

**MATERIALS AND METHODS:**

In this retrospective cohort study, 43 women underwent bilateral tubal ligation via vNOTES at the Iğdır Dr. Nevruz Erez State Hospital. Twenty patients received PBC with 10 ml of 0.5% bupivacaine injected at the 3 and 9 o’clock cervicovaginal junction, whereas 23 women served as controls. Outcomes included visual analog scale (VAS) pain scores at 1, 6 and 24 hours postoperatively, intra- and postoperative opioid use, and Female Sexual Function Index (FSFI) scores preoperatively and 1 month after surgery.

**RESULT:**

PBC significantly lowered 24-hour VAS scores (mean [SD], 3 vs 4; *P* = 0.02) and intraoperative opioid requirement (mean [SD], 0 vs 10 mg morphine equivalent; *P* = 0.01). Total postoperative analgesic consumption and length of hospital stay were comparable. FSFI scores 1 month after surgery were higher in the PBC group than the controls (mean [SD], 206 vs 14.5 respectively; *P* = 0.001), indicating better short-term sexual function recovery.

**CONCLUSION:**

Incorporating PBC into vNOTES enhances early pain control and decreases intraoperative opioid use without delaying discharge, while favorably influencing short-term sexual function. PBC is a simple, effective adjunct that aligns with ERAS goals in minimally-invasive gynecologic surgery.

## INTRODUCTION

Tubal sterilization is a common form of permanent contraception. In the United States, roughly 700 000 women undergo bilateral tubal ligation (BTL) annually, with about half of the procedures conducted during the peri- or postpartum period, and the remainder, as interval laparoscopic procedures.^1^ Among women aged 20–49 years currently using contraception, female sterilization accounts for roughly 18% of all contraceptive methods.^2^ Traditionally, interval sterilization has been achieved via laparoscopic BTL, but in recent years, vaginal natural orifice transluminal endoscopic surgery (vNOTES) has become an alternative minimally-invasive approach.^3^

NOTES is a major milestone in minimally-invasive surgery.^4^ Its transvaginal variant (vNOTES) employs the posterior vaginal fornix as a natural gateway to the peritoneal cavity, allowing laparoscopic the performance of procedures without abdominal incisions. Numerous prospective and retrospective studies have confirmed the feasibility and safety of vNOTES, consistently reporting less immediate postoperative pain, faster functional recovery, and scar-free cosmesis—advantages that make genuine day-case surgery feasible.^5^;^6^;^7^;^8^;^9^;^10^;^11^;^12^;^13^;^14^ Therefore, vNOTES is particularly attractive for BTL as a true day-case sterilization procedure.

Despite these benefits, postoperative pain—especially vaginal or pelvic discomfort—remains a concern after vNOTES.^15^ To address this, a paracervical block (PBC) can be employed by injecting a long-acting local anesthetic (such as 0.5% bupivacaine) at the cervical junction to block the pelvic nerve plexus. PBC has a long history in obstetric and gynecologic practice, and has been shown in randomized trials to significantly reduce immediate postoperative pain after laparoscopic gynecologic surgeries.^16^;^17^ However, its use specifically during vNOTES sterilization has not been reported.

In this retrospective study, we compared cases of bilateral tubal ligation via vNOTES performed with and without the addition of preoperative PBC.

## AIM

Our primary aim was to evaluate whether PBC reduced intraoperative and postoperative opioid and other analgesic requirements, lowered early postoperative pain scores, and facilitated discharge without impairing recovery or sexual function. By integrating these outcomes, the study aimed to contribute to the growing body of literature on pain control, recovery, and discharge optimization in minimally-invasive gynecologic sterilization.

## MATERIALS AND METHODS

### Study population

This study included women aged 18 years or older who requested permanent surgical sterilization and had no pelvic organ prolapse greater than stage 2, as assessed by the Pelvic Organ Prolapse Quantification system. Exclusion criteria were: current pregnancy, active pelvic infection, suspected pelvic malignancy, history of rectovaginal endometriosis, pelvic abscess, or a lack of prior vaginal intercourse.^18^

In line with the institutional guidelines, bilateral salpingectomy was recommended over BTL for all patients. However, in accordance with patient choice, those who declined salpingectomy and opted for tubal ligation were included in this study.

The participants were divided into 2 cohorts according to whether intraoperative PBC was employed: the PBC+vNOTES and the vNOTES groups (controls). For both cohorts, baseline demographic and clinical variables including age, height, weight, body mass index (BMI), parity, and any history of prior abdominal or pelvic surgery were meticulously recorded.

This retrospective cohort used consecutive sampling. All women who met the eligibility criteria during the predefined study period (September 20, 2023 to December 1, 2024) were included. This approach yielded a final sample of 43 patients, with 20 women in the PBC+vNOTES group and 23 participants in the control group. No a priori effect-size assumption or formal sample size calculation was used to determine enrollment.

### Surgical procedure

All surgical procedures were performed by gynecologic surgeons with established proficiency in both conventional laparoscopy and vNOTES. Preoperatively, each patient received a single dose of 2 g of intravenous cefazolin as antibiotic prophylaxis, in accordance with the institutional infection control guidelines.

All procedures were conducted under general anesthesia with the patient placed in the lithotomy position and tilted at the Trendelenburg angle of 15 ° to 30 °. No patients received any form of preoperative analgesia, such as nonopioid or opioid adjuncts. A standardized anesthesiology protocol was used for all women. None of the patients underwent simultaneous epidural analgesia. Anesthesia was induced via an intravenous injection of lidocaine (1 mg/kg), propofol (2 mg/kg), fentanyl (1 μg/kg), and rocuronium (0.6 mg/kg). After endotracheal intubation, anesthesia was maintained with sevoflurane at 1 MAC in a mixture of 50% oxygen and 50% air, and the continuous infusion of remifentanil (2–15 μg/kg/h) to maintain hemodynamic variation within 20% of baseline. Alveolar recruitment maneuvers (30 cm H_2_O positive airway pressure sustained for 30 s) were performed on every patient at the end of the surgery. Special care was taken to maintain normothermia and to control glycemia. A regimen of fluids and electrolytes was established to ensure normovolemia. Postoperative instructions included abstaining from sexual intercourse for at least 2 weeks to facilitate vaginal healing and reduce the risk of infection.

### Vaginal natural orifice transluminal endoscopic surgery group

A 2.5 cm posterior colpotomy was performed by incising the posterior vaginal mucosa to access the pouch of Douglas. A GelPOINT V-Path transvaginal access platform (Applied Medical, Rancho Santa Margarita, California, United States) was inserted to establish working channels. A standard 30 ° 10-mm laparoscope and conventional reusable laparoscopic instruments, including LigaSure (Medtronic, Minneapolis, Minnesota, United States) and graspers, were used to perform bilateral tubal ligation.^18^ Postoperatively, all patients in this group received standardized analgesia with 1 g of paracetamol and 100 mg of tramadol. Pain was assessed with a visual analogue scale (VAS) 1 hour postoperatively. The patients whose VAS score was equal to or higher than 4 received additional 100 mg of tramadol as rescue analgesia. Additionally, all women were administered 1 g of paracetamol routinely every 8 hours during the postoperative period.

### Vaginal natural orifice transluminal endoscopic surgery and paracervical block group

After induction of general anesthesia and sterile preparation, PCB was administered by a surgeon by infiltrating 0.5% bupivacaine in 2 equal aliquots (10 ml in total) at the 3 and 9 o’clock positions of the cervicovaginal junction.^19^ No block-related injection or surgical complications were observed in any patient. Once adequate diffusion had been allowed, all subsequent operative steps, including creation of the posterior colpotomy, placement of the GelPOINT V-Path transvaginal access platform, and completion of BTL with a 30 ° 10-mm laparoscope and standard reusable instruments (LigaSure and graspers), were identical to the protocol used in the vNOTES group. Postoperatively, all patients in this group received 1 g of paracetamol and 100 mg of tramadol only as analgesia. Pain was assessed with the VAS 1 hour after surgery. The patients whose VAS score was equal to or higher than 4 received additional 100 mg of tramadol as rescue analgesia. Additionally, all women were administered 1 g of paracetamol routinely every 8 hours during the postoperative period.

The Female Sexual Function Index (FSFI) questionnaire was used to assess sexual function. It was self-administered by the patients in written form during the preoperative period (1 week prior to surgery) and 1 month postoperatively. The questionnaire consists of 19 questions covering 6 domains: desire, arousal, lubrication, orgasm, satisfaction, and pain. The results were scored according to the original FSFI scoring method.

Postoperative pain intensity was assessed using the VAS, a validated and widely used tool for measuring acute pain intensity on a continuous scale. The use of these validated instruments—the VAS for pain and the FSFI for sexual function—ensured reliability and validity of our outcome assessments.

### Statistical analysis

All statistical analyses were conducted using IBM SPSS Statistics software, version 26.0 (IBM Corp., Armonk, New York, United States). Descriptive statistics were presented as mean (SD), frequencies, and percentages. The normality of distribution for continuous variables was assessed using the Shapiro–Wilk test (for n <⁠50). For normally distributed continuous variables, comparisons between the groups were performed using the independent samples *t* test; for non-normally distributed variables, the Mann–Whitney test was employed. Categorical variables were compared using the χ² test. A *P* value below 0.05 was considered significant.

### Ethics

This study was designed as a retrospective observational study conducted at the Gynecology and Obstetrics Outpatient Clinic of the Iğdır Dr. Nevruz Erez State Hospital between September 20, 2023 and December 1, 2024. It was approved by the Kayseri City Hospital Non-Interventional Clinical Research Ethics Committee (472). All procedures performed in this study involving human participants were conducted in accordance with the ethical standards of the institutional research committee, the 1964 Helsinki Declaration, and its later amendments, or comparable ethical standards.

## RESULTS

In this retrospective cohort study, we compared bilateral tubal ligation via vNOTES with PBC (PBC+vNOTES group) and without PBC (vNOTES group). A total of 43 women met the inclusion criteria, with 20 patients assigned to the PBC+vNOTES group and 23, to the vNOTES group.

Baseline characteristics were well balanced between both cohorts: mean (SD) age was 35 (5) years in the PBC+vNOTES group and 35 (4) years in the vNOTES group (*P* = 0.41). No significant differences were detected in terms of BMI, history of prior abdominal / pelvic surgery. This demographic homogeneity strengthens the internal validity of our efficacy and safety comparisons across the pre-, intra- and postoperative periods. Full baseline data are presented in Table 1.

**Table 1:** Demographic characteristics of the patients

Parameter		All participants (n = 43)	vNOTES (n = 23)	PBC+vNOTES (n = 20)	*P* value
Age, y		35 (4)	35 (5)	35 (4)	0.41
Height, cm		159 (6)	158 (6)	160 (6)	0.28
Weight, kg		73 (11)	73 (10)	73 (11)	>0.99
BMI, kg/m^2^		28.9 (4.9)	29.2 (4.6)	28.5 (4.8)	0.48
Previous surgery	No	10 (23)	16 (70)	17 (85)	0.29
	Yes	33 (77)	7 (30)	3 (15)	
Number of vaginal deliveries		4 (2)	4 (2)	4 (4)	0.9

Data are presented as mean (SD) or numbers (percentages).

Abbreviations: BMI, body mass index; PBC, paracervical block; vNOTES, vaginal natural orifice transluminal endoscopic surgery

Intraoperative findings are illustrated in Table 2. Operative time was comparable between the groups (*P* = 0.14), whereas both blood loss (*P* = 0.001) and total intraoperative opioid consumption (*P* = 0.01) were lower in the PBC+vNOTES group.

**Table 2:** Intraoperative outcomes and hemodynamic parameters

Parameter	All participants (n = 43)	vNOTES (n = 23)	PBC+vNOTES (n = 20)	*P* value
Duration of surgery, min	25 (10)	25 (15)	23 (5)	0.14
Intraoperative blood loss*, *ml	20 (20)	30 (30)	20 (10)	0.001
Total intraoperative opioid consumption*, *μg	360 (180)	420 (360)	330 (130)	0.01

Data are presented as mean (SD).

Abbreviations: see Table 1

Table 3 outlines the postoperative outcomes. Pain scores at 1 hour and 6 hours postoperatively were similar between the PBC+vNOTES and vNOTES groups (*P* = 0.85 and *P* = 0.92, respectively), but the 24-hour VAS score was lower in the PBC+vNOTES cohort (*P* = 0.02). Both groups had a mean (SD) hospital stay of 1 (0) day (*P* = 0.58), and there were no intergroup differences in the proportion of patients requiring additional postoperative analgesia (*P* = 0.64). Overall, PBC improved late-first-day pain without affecting discharge timing or rescue analgesic use.

**Table 3:** Postoperative pain and recovery outcomes

Parameter		All participants (n = 43)	vNOTES (n = 23)	PBC+vNOTES (n = 20)	*P* value
VAS score, points	1 hour postoperatively	5 (1)	5 (2)	5 (1)	0.85
	6 hours postoperatively	4 (2)	4 (2)	5 (2)	0.92
	24 hours postoperatively	2 (2)	3 (3)	2 (3)	0.02
Length of hospital stay, d		1 (0)	1 (0)	1 (0)	0.58
Postoperative analgesic requirement	Yes	10 (23)	6 (26)	4 (20)	0.64
	No	33 (77)	17 (74)	18 (80)	

Data are presented as mean (SD) or numbers (percentages).

Abbreviations: VAS, visual analog scale; others, see Table 1

Sexual function outcomes are shown in Table 4. Preoperative FSFI scores did not differ between the groups (*P* = 0.08), but at 1 month after surgery, the PBC+vNOTES group achieved higher FSFI values (*P* = 0.001), indicating superior short-term recovery of sexual function following PBC.

**Table 4:** Comparison of female sexual function index scores before and after surgery

Parameter	All participants (n = 43)	vNOTES (n = 23)	PBC+vNOTES (n = 20)	*P* value
Preoperative FSFI	22 (7)	21 (7)	24 (6)	0.08
Postoperative FSFI	17 (6)	14 (5)	20 (6)	0.001

Data are expressed as mean (SD).

Abbreviations: FSFI, female sexual function index; others, see Table 1

## DISCUSSION

Our study compared conventional vNOTES BTL with vNOTES performed under additional PBC. The PBC+vNOTES group demonstrated a marked reduction in intraoperative opioid use, lower 24-hour postoperative VAS pain scores, and higher FSFI scores at 1 month after surgery.

PBC was clearly associated with improved analgesia: 24-hour VAS scores were considerably lower in the PBC+vNOTES patients than in the controls, confirming that the addition of a long-acting local anesthetic provides superior pain control. This finding is consistent with the body of evidence showing that vNOTES is inherently less painful than standard laparoscopy^20^ and suggests that PBC amplifies this advantage. Comparable results were reported in a recent randomized trial of laparoscopic hysterectomy, where PBC with 0.5% bupivacaine significantly reduced early postoperative VAS scores and analgesic demand.^21^ Thus, PBC appears to confer an additional benefit even on the already low-pain profile of vNOTES.

Optimization of the transvaginal access platform may influence the efficiency and safety of vNOTES. In our protocol, we used the GelPOINT V-Path system. Notably, ^22^ compared the GelPOINT and glove techniques in vNOTES hysterectomy (n = 60) and reported shorter operative time and fewer complications with GelPOINT, albeit at higher material cost, while both techniques were safe and effective.

Despite the clear improvement in pain scores, total postoperative opioid / analgesic consumption did not differ between the groups. This may reflect our routine multimodal analgesic protocol, a combination of paracetamol and tramadol, which could mask differences in rescue requirements. Nevertheless, the PBC+vNOTES cohort displayed a downward trend in postoperative analgesic demand, and their intraoperative opioid requirement was markedly lower, indicating that the block attenuated nociceptive input during surgery. A recent systematic review also showed that local anesthetic blocks reduced 24-hour opioid consumption after laparoscopic or vaginal hysterectomy.^23^ Although our sample size was limited, the cumulative evidence supports an opioid-sparing effect of PBC, in line with the enhanced recovery after surgery (ERAS) objectives, to minimize opioid exposure and its adverse effects.

Future studies might consider employing more sensitive measures of analgesic usage or larger sample sizes to further clarify the relationship between PBC use and lower demand for opioids, which carries important clinical benefits: opioids, although effective, can delay recovery by causing sedation, nausea, and ileus. By reducing opioid requirements, PBC may facilitate earlier mobilization and improve patient satisfaction in ambulatory surgery. In our series, all patients were discharged either on the day of surgery or within 24 hours, consistent with the day-case profile of vNOTES. Effective pain relief provided by PBC likely strengthened this advantage by reducing breakthrough pain and rescue medication needs, thereby supporting an optimal ERAS pathway.

The significantly lower intraoperative blood loss observed in the PBC+vNOTES group can be explained by 2 complementary mechanisms: the intrinsic vasoconstrictive properties of bupivacaine and improved hemodynamic stability due to reduced nociceptive stress responses. Clinically, this reduction in bleeding may enhance surgical visibility, reduce perioperative complications, and potentially shorten operative time. Although the observed absolute difference was modest, its cumulative clinical benefit might be substantial, particularly in patients at a higher risk of intraoperative bleeding or those undergoing more extensive procedures. Further investigation in larger prospective trials is warranted to confirm these potential clinical advantages and clarify their practical significance.

Equally noteworthy, PBC had no adverse impact on female sexual function—1-month FSFI scores were considerably higher in the PBC+vNOTES group. Both cohorts experienced a modest decline relative to baseline, expected during early recovery, yet the PBC+vNOTES group maintained a better score, suggesting faster restoration of sexual function when postoperative pain is better controlled. By alleviating discomfort, PBC may mitigate short-term dyspareunia or reduced desire and help patients reach the favorable long-term sexual outcomes reported after sterilization.

Although our findings are encouraging, several limitations must be acknowledged. First, since the investigation was single-center, retrospective, and nonrandomized, unmeasured confounding and selection bias between the patients who did and did not receive PBC cannot be excluded. The relatively small sample size (n = 43) further reduces the statistical power of the analyses. Neither the surgical nor the anesthesia teams, nor the patients were blinded to PBC administration; consequently, subjective pain reporting and requests for analgesia may have been influenced by perception bias. Pain assessment was confined to the first 24 hours after surgery, and FSF was evaluated only at the first postoperative month, leaving medium- and long-term outcomes unmeasured. These factors should be considered when interpreting our results.

Future research should include longer follow-up periods, potentially 6 months or more, to comprehensively assess the persistence of analgesic benefits and to detect any delayed effects on sexual function, patient satisfaction, or quality of life.

Another limitation of this study is the relatively small sample size together with its retrospective, nonrandomized design. These factors inherently limit statistical power and increase the risk that selection bias and unmeasured confounders may have influenced the results. A further key limitation is that our primary analyses were univariable. Given the small sample and limited number of events, multivariable models would likely have been overfitted and yielded unstable estimates, so no adjustment was performed. To strengthen the evidence base regarding the efficacy and safety of PBC during vNOTES BTL, larger-scale, prospective randomized controlled trials are needed. Such studies would reduce bias, provide higher-quality evidence, and enable more definitive conclusions.

## CONCLUSIONS

In summary, adding PBC to vNOTES provides meaningful benefits, such as considerably reduced intraoperative blood loss, superior early analgesia, limited opioid exposure, and improved short-term sexual function without compromising surgical outcomes. These gains are particularly valuable for fast-track, minimally-invasive gynecologic procedures in which optimal pain control supports rapid discharge. Although the present retrospective, single-center study is limited by its small sample size, it is one of the first to evaluate PBC in the vNOTES setting and offers new evidence supporting the integration of regional anesthesia techniques into vNOTES protocols. Larger prospective trials with longer follow-up are warranted to confirm these findings and elucidate long-term effects; however, current data support PBC as a valuable adjunct for optimizing perioperative hemostasis, pain control, and recovery in minimally-invasive sterilization surgery.
